# Optimisation of UV irradiation as a binding site conserving method for crosslinking collagen-based scaffolds

**DOI:** 10.1007/s10856-015-5627-8

**Published:** 2015-12-16

**Authors:** Natalia Davidenko, Daniel V. Bax, Carlos F. Schuster, Richard W. Farndale, Samir W. Hamaia, Serena M. Best, Ruth E. Cameron

**Affiliations:** Department of Materials Science and Metallurgy, University of Cambridge, 27 Charles Babbage Road, Cambridge, CB3 0FS UK; Department of Biochemistry, University of Cambridge, Downing Site, Cambridge, CB2 1QW UK

## Abstract

Short wavelength (λ = 254 nm) UV irradiation was evaluated over a range of intensities (0.06 to 0.96 J/cm^2^) as a means of cross-linking collagen- and gelatin-based scaffolds, to tailor their material characteristics whilst retaining biological functionality. Zero-link carbodiimide treatments are commonly applied to collagen-based materials, forming cross-links from carboxylate anions (for example the acidic E of GFOGER) that are an essential part of integrin binding sites on collagen. Cross-linking these amino acids therefore disrupts the bioactivity of collagen. In contrast, UV irradiation forms bonds from less important aromatic tyrosine and phenylalanine residues. We therefore hypothesised that UV cross-linking would not compromise collagen cell reactivity. Here, highly porous (~99 %) isotropic, collagen-based scaffolds were produced via ice-templating. A series of scaffolds (pore diameters ranging from 130–260 μm) with ascending stability in water was made from gelatin, two different sources of collagen I, or blends of these materials. Glucose, known to aid UV crosslinking of collagen, was added to some lower-stability formulations. These scaffolds were exposed to different doses of UV irradiation, and the scaffold morphology, dissolution stability in water, resistance to compression and cell reactivity was assessed. Stabilisation in aqueous media varied with both the nature of the collagen-based material employed and the UV intensity. Scaffolds made from the most stable materials showed the greatest stability after irradiation, although the levels of cross-linking in all cases were relatively low. Scaffolds made from pure collagen from the two different sources showed different optimum levels of irradiation, suggesting altered balance between stabilisation from cross-linking and destabilisation from denaturation. The introduction of glucose into the scaffold enhanced the efficacy of UV cross-linking. Finally, as hypothesized, cell attachment, spreading and proliferation on collagen materials were unaffected by UV cross-linking. UV irradiation may therefore be used to provide relatively low level cross-linking of collagen without loss of biological functionality.

**Electronic supplementary material:**

The online version of this article (doi:10.1007/s10856-015-5627-8) contains supplementary material, which is available to authorized users.

## Introduction

In the development of tissue engineering (TE) devices, particularly for soft tissue repair and regeneration, scaffolds composed of proteins [especially of those found in the extracellular matrix (ECM)] present a promising starting point. The benefits of such biomaterials are their natural origin and their similar protein composition and mechanical properties to native tissue. They also provide appropriate binding motifs for cell attachment, proliferation and maintenance of phenotype [1–3]. In this work collagen (Col) and gelatin (Gel) were selected as two main components for the development of 3D scaffolds with controlled material characteristics (morphology, dissolution kinetics, swelling capacity and mechanics) for their potential use for soft tissue regeneration. In particular, this study has focused on the use of ultraviolet irradiation (UV) at short wavelength (254 nm) to cross-link collagen-based scaffolds.

Collagen, being the main protein of ECM, has been widely used in different TE applications [1, 4–6] with particular emphasis on soft tissue regeneration, such as cardiac repair [6–8]. It has been shown that collagen-based scaffolds not only support the native tissue during repair, but also play an important role in providing essential signals to influence cell activity [9]. Gelatin, a partially denatured collagen, has also been employed alone or in combination with other biopolymers in a variety of biomedical devices, for example, as meshes to treat heart defects [10–12]. Gelatin is easier to extract and prepare than collagen and thus is much cheaper and can be produced in larger quantities [13–16]. The denaturation process results not only in different physical and chemical properties of Gel compared to Col, but also in altering the available cell-binding sites on Gel. In essence these change from the triple-helical cell binding motifs such as GFOGER on Col to linear, or flexible RGD motifs on Gel [17–19]. As such, the combination of Col and Gel may allow the scaffold properties and cell interactions to be conveniently tailored.

Protein-based scaffolds often need to be crosslinked (XL) to provide the required structural stability and mechanical strength for use in different biological environments [20–26]. For this purpose, a variety of chemical [26–32] or physical [33–37] methods can be employed. One such chemical treatment for collagen-based biomaterials is soluble carbodiimide, 1-ethyl-3-(3-dimethylaminopropyl)-carbodiimide hydrochloride (EDC), in the presence of *N*-hydroxy-succinimide (NHS). This treatment is very efficient and nontoxic since neither EDC nor NHS take part in linkage formation and any by-products can be easily removed by [26, 31, 32, 38]. Despite this, EDC-promoted bonding presents a significant drawback as it uses free primary amino groups (on lysine residues) and carboxylate anions (on glutamate or aspartate residues) for cross-linking. Many of these same amino acid side chains, for example the acidic E of GFOGER, represent essential cell binding sites on collagen-type materials and so EDC-promoted crosslinking can impinge on bioactivity.

To overcome this disadvantage, this study employed UV for scaffold cross-linking. By contrast to EDC-promoted linkage, UV irradiation forms bonds between the nuclei of aromatic residues, such as those of tyrosine and phenylalanine [34, 39, 40]. Therefore UV irradiation offers the potential to link protein molecules, and so provide bonding within polypeptide chains, without utilising the acidic and basic side chains that are important cell recognition sites. Furthermore, UV treatment benefits from avoiding the need for toxic chemicals, is easy to perform, and is environmentally friendly. Another attraction of UV as a crosslinking methodology is its strong sterilisation effect, as UV light at 254 nm can effectively kill microorganisms by destroying their nucleic acids, leading to disruption of their DNA [41, 42]. Indeed UV light has been frequently used as a tool for obtaining materials for specific biomedical purposes [34, 35, 37, 43, 44]. During UV exposure of collagen, two processes, crosslinking and UV-induced denaturation, oppose and compete. It is the balance of these two processes that ultimately influences the mechanics and degradation behaviour of irradiated samples [34, 35]. This complexity makes it difficult to achieve precise control over the degree of transformation and to predict the outcome of UV treatment on collagen-based assemblies that possess different macromolecular order. As the processes involved in the irradiation of biomaterials by UV light are not fully understood, the current systematic study was designed to elucidate the properties of collagen- and gelatin-based scaffolds exposed to UV light of different intensities. Alongside this, we were interested in evaluating the possible synergetic effect of glucose and UV in crosslinking Col and Gel-based materials, as glucose inclusion in collagen films can increase the crosslinking efficiency of UV light [35]. This effect of glucose inclusion results, on the one hand from the formation of additional, glucose-derived, bonds in the polypeptide chains and on the other hand by limiting UV-induced denaturation of collagen, by preventing local unravelling of its triple helical structure.

In this study we made a series of scaffolds with ascending stability in water by using gelatin and two different sources of Col I, as well as blends of these materials with Gel. This allowed us to address the possible influence of the source material stability on the resultant scaffolds under UV irradiation. Col-based scaffolds were produced using two types of microfibrillar Col I obtained from different providers which result in scaffolds with different stability: collagen supplied by Sigma (S) formed scaffolds with a higher stability than collagen supplied by Devro (D). Both collagens were of bovine origin but derived from different tissues: Col(S) was obtained from achilles tendon while Col(D) was extracted from the epidermal layer of skin. Collagen assembly and network formation are tissue specific with each tissue containing different macromolecular triple-helical structures (fibril lengths, width, entanglement, etc.). Additionally different purification processes between these two sources may affect the physical properties of the resulting collagen differently. The series Col(S) > Col(D) > Gel therefore represent a series of descending stability, with blends of Col(S)–Gel and Col(D)–Gel providing intermediate properties. This enabled us to assess the influence of the source collagen on the final properties of the scaffolds obtained. Using this, in combination with the UV cross-linking conditions, may allow for tailored scaffold properties to more closely match those of the tissues they aim to repair.

The biological performance of collagenous materials before and after UV irradiation was also assessed in this work by studying cell adhesion, spreading and proliferation on thin films. It is known that in 3D matrices cells adhere to and spread on the struts [21, 45]. Therefore by modelling these struts as thin 2D films, the interaction of cells with the biomaterials can be investigated, avoiding the complications of 3D structure. Human fibrosarcoma (HT1080) and human platelets were selected for this biological study. Both utilize integrin α2β1 as a major collagen binding receptor [46, 47]. Integrins are a class of heterodimeric cell surface receptor, composed of one alpha subunit and one beta subunit that bind to the ECM surrounding a cell. There are four collagen binding integrins, namely α1β1, α2β1, α10β1 and α11β1. The collagen binding activity of these integrins is dependent upon the presence of divalent cations such as magnesium, making EDTA chelation of cations a convenient test for integrin-based cell activity. Alongside integrin α2β1, cells can bind to collagen via cell surface glycoproteins such as GPVI on platelets [46, 48] and DDR-1 and -2 on HT1080s. It is well documented that the native type I collagen triple-helical conformation plays a crucial role for the recognition of integrins α1β1 and α2β1 through GXOGEX’ motifs [46–49]. By contrast, denaturation of collagen to gelatin leads to the loss of the triple helical collagen conformation and so reduced cell adhesion to the GFOGER sites. Instead the primary cell binding motif in gelatin is RGD which ligates other classes of integrin, such as α5β1 and αvβ3 [18]. Therefore investigating the cell binding activity of Col films, with and without UV treatment, allowed measurement of potential UV ablation of cell-binding motifs in the collagen scaffold.

## Materials and methods

### Materials

Fibrous collagen I derived from bovine dermal tissue was purchased from Devro Medical Ltd., Australia, and from bovine achilles tendon was obtained from Sigma–Aldrich Co. Ltd., UK. Gelatin (type B from bovine skin, Gel), acetic acid (2 M), EDC and NHS were purchased from Sigma-Aldrich (UK). Dulbecco Modified Eagles Medium (DMEM, Gibco), phosphate buffered saline (PBS, Gibco), Foetal Calf Serum, penicillin and streptomycin were purchased from Invitrogen Life Sciences (UK). Other commercially available reagents were all analytical grade.

### Scaffold and film preparation

Protein scaffolds (Col, Gel and Col/Gel = 50 % w/w.) were obtained by a freeze-drying. Briefly, collagen was swollen in 0.05 M acetic acid at 4 °C overnight to produce a 1 % (w/v) protein suspension. The resulting suspension was homogenised on ice for 30 min at 13500 rpm using an Ultra-Turrax VD125 (VWR International Ltd., UK). Air bubbles were removed from the suspension by centrifuging at 2500 rpm for 5 min (Hermle Z300, Labortechnik, Germany). Gelatin solution, 1 % (w/v), was prepared by dissolving gelatin in 0.05 M acetic acid at 37–45 °C with stirring for 1 h. The solution was then cooled to room temperature with stirring. Collagen–gelatin 1:1(wt.) suspension was prepared by mixing a gelatin solution with a collagen slurry (both 1 % w/v), homogenising for 15 min and centrifuging at the same conditions as Col suspensions. All suspensions were then poured into silicone rubber trays (Lakeland, UK), to ensure ease of removal, and freeze-dried in a VirTis adVantage bench-top freeze-drier (Biopharma Process Systems, UK) using a cycle adapted from our previous work [3, 38]. A constant cooling rate of 0.9 °C/min to a final freezing temperature of −26 °C was used. The temperature was then held constant at −26 °C for 90 min. The ice phase was sublimed for 20 h under a vacuum of less than 100 mTorr.

Scaffolds with glucose (G) at protein/G ratio = 4.65(wt/wt) were produced using the same freeze-drying protocol as described above. Glucose was added to Col suspension (for Col-containing scaffolds) or to Gel solution before mixing.

For cell studies films of ~8 µm of thickness were prepared by drying the corresponding 0.5 % suspension of protein (Col(S), Col(S)–Gel and Gel) directly in Immulon-2HB 96-well plates (100 μl of slurry/well) for 48 h in a laminar flow cabinet.

### Scaffold crosslinking

Protein scaffolds (Col, Gel and Col/Gel = 50/50) were cross-linked by exposure to UV light using UVP UV Crosslinker (CL-1000 Series, Ultra-Violet Products Ltd., UK). UV irradiation with λ = 254 nm; t = 30 min (each side of scaffold sample) at different intensities (from 0.06 to 0.96 J/cm^2^) was used to assess the possible effect of the intensity of UV light on the degree of cross-linking. An irradiation time of 30 min was selected on the bases of reports suggesting that after 30 min the cross-linking density does not increase and that prolonged UV exposure contributes to denaturation of collagen molecules [34]. To assess the possible synergetic effect of glucose and UV irradiation scaffolds with glucose were cross-linked by UV light at 0.42 J/cm^2^ for 30 min (each side).

Films in 96-well pates were irradiated with UV light for 30 min at 0.42 or 0.96 J/cm^2^ for 30 min.

### Morphology and porosity

#### Morphology

Scanning electron microscopy (SEM) (JEOL 5800) was used to analyse the internal architecture of scaffolds with and without UV exposure. Longitudinal and transverse cross-sections were cut from different sections of the scaffold sheet, mounted on stubs and sputter coated with a layer of platinum for observation at 10 keV at various levels of magnification. Images were used to compare the characteristics of scaffold porous structures (homogeneity, interconnectivity, etc.) and to determine their pore sizes by manually measuring different pore zones (at least 30 pores randomly chosen from different microphotographs of each sample).

#### Porosity

The scaffold porosity was measured as the percentage of void space in a 3D sponge. This parameter was determined from the relative density of scaffolds, ρ*/ρ_s_, using the following equation [50]:

$${\text{Porosity }}\left( \% \right) \, = \, \left( {1 \, - \, \uprho */\uprho_{\text{s}} } \right) \, \times 100)$$

where ρ* is relative density and ρ is known dry density.

Relative density was calculated from the measured dry density of the scaffold post freeze-drying (ρ* = m/V; where m is the mass and V is the volume of the 3D matrix) and the known dry density of solid collagen (gelatin) (ρ_s_), in this case 1.3 g/ml [28].

### Dissolution in distilled water

Dissolution studies were carried out at 37 °C in distilled water. Specimens of approximately 5 mg were accurately weighed (Mb) and then incubated in 5 ml of distilled water at 37 °C for up to 28 days. At each selected time point the samples were removed from the water, dried until constant weight is achieved between drying steps and then weighed (Ma). The percentage mass loss was calculated as shown:

$${\text{Mass loss }}\left( \% \right) \, = \, 100 \, \times \, \left\{ {\left( {{\text{Mb}} - {\text{Ma}}} \right)/{\text{Mb}}} \right\}$$

Tests were repeated with four parallel samples for each condition.

### Amine group content and degree of cross-linking

The content of free primary amine groups present was determined using a 2,4,6-trinitro-benzene-sulfonic acid (TNBS) assay with a protocol similar to those reported by Sashidar et al. and Ofner et al. [51, 52]. To each sample (2–4 mg), 0.5 ml of a 4 % (w/v) NaHCO_3_ solution and 0.5 ml of a freshly prepared solution of 0.05 % (w/v) TNBS were added. After 2 h at 40 °C, 1.5 ml of 6 M HCl was added and the samples were hydrolysed at 60 °C for 90 min. The reaction mixture was diluted with distilled water (2.5 ml), cooled to room temperature, and the A_320_ was measured using a Fluostar Optima spectrophotometer. Controls (blank samples) were prepared using the same procedure, except that HCl was added, prior to introducing the TNBS solution, to prohibit any reaction of TNBS with the amine groups. The blank sample value was subtracted from each sample absorbance. The absorbance was correlated to the concentration of free amine groups using a calibration curve obtained with glycine in an aqueous NaHCO_3_ solution. Three replicates were used for each condition and the number of amino groups per 1000 residues calculated. Results are expressed in the figures as mean ± standard error measurement.

### Mechanical testing

Compressive stress–stain analysis of the scaffolds was performed in the wet stage using a mechanical Hounsfield tester, equipped with a 5 N load cell. Cylindrical specimens, of 8 mm diameter and 6–8 mm thickness, were cut from the scaffolds and hydrated in deionised water at room temperature for 1 h prior to testing. All compression tests were performed perpendicular to the plane of the scaffold disc at a crosshead speed of 5 mm/min. The linear elastic (Young’s) modulus was obtained via linear regression of the initial linear region of the stress–strain curve. Five replica tests were made for each scaffold condition.

### Glucose release from scaffolds

Glucose release from scaffolds was assessed using an enzymatic assay based on the activity of glucose oxidase/peroxidase/*o*-dianisidine which forms a coloured (pink, A_540_) product (oxidized 0-dianisidine) [53]. The intensity of this colour is proportional to the original glucose content. For this assay, samples of approximately 5–10 mg of scaffolds with glucose (before and after UV irradiation with intensity of 0.42 J/cm^2^) were weighed and placed in 4 ml of deionised water for 1 and 24 h. Samples were tested in triplicate. Samples without glucose were used as a negative control. At each time point (1 and 24 h) samples were removed from the water and placed in 4 ml of fresh deionised water for washing. This washing solution was combined with the first water incubation solution extract and the resultant 8 ml of solution was further diluted to obtain a final volume of 10 ml. 100 μl of this solution was diluted again (four times) in an Eppendorf tube and mixed by vortexing. 20 μl of this solution was added in triplicate to wells in 96-well plate. Alongside these test conditions, d-glucose standards (1.0 mg/ml in 0.1 % benzoic acid) were diluted to different concentrations and added to empty wells of the 96-well plate to allow calibration against known standards. The assay reagent was prepared from o-dianisidine reagent and the mixture of glucose oxidase/peroxidase, according to test instruction (assay kit GAGO-20, Sigma). 40 μl of this assay reagent were pipetted to each well and then 96-well plate was placed in incubator at 37 °C for 30 min. Then 40 μl of 12 M H_2_SO_4_ were added to stop the reaction. The absorbance at 540 nm was measured using a Fluostar Optima spectrophotometer.

### Cells/platelets reactivity on collagen films

HT1080 cells derived from a human fibrosarcoma were obtained from the European Collection of Animal Cell Cultures, Porton Down, UK. HT1080s were maintained in a humidified incubator with 5 % CO_2_ at 37 °C in DMEM (Gibco), containing 10 % fetal bovine serum (Gibco) and 1 % streptavidin/penicillin (Life Technologies). Prior to cell adhesion/spreading experiments, HT1080 s were detached from the cell culture flasks with 0.05 % trypsin/0.02 % EDTA (GE Healthcare), washed and re-suspended in serum free DMEM. Platelets were obtained from human platelet rich plasma provided by the National Health Service Blood and Transplants authority in accordance with the Declaration of Helsinki. Platelets were prepared by centrifugation for 15 min, 240 g, the pellet discarded and 1 μl of Prostaglandin E_1_ (100 μg/ml in ethanol) added per ml of platelet supernatant. The platelet suspension was centrifuged at 640 g for 10 min and the platelet pellet resuspended in tyrodes buffer (140 mM NaCl, 5.6 mM Glucose, 2 mM MgCl_2_, 0.4 mM NaH_2_PO_4_, 12 mM NaHCO_3_, 2.7 mM KCl, 10 mM HEPES pH 7.4) to a density of 1 × 10^8^/ml.

#### Platelet adhesion assay

Platelet adhesion in the presence of Mg^+2^ (integrin mediated) and EDTA (non-specific) was determined by a colorimetric assay based on the detection of Phosphatase activity released through platelet lysis [46]. Before platelet incubation, non-specific adsorption to the scaffold films was blocked with 200 μl of bovine serum albumin (BSA, 5 % (w/v) in PBS) for 60 min, and then washed three times with 200 μl of PBS. 100 μl of platelet suspension at 1 × 10^8^ pl/ml in Tyrodes buffer (140 mM NaCl, 5.6 mM glucose, 2 mM MgCl_2_, 0.4 mM NaH_2_PO_4_, 12 mM NaHCO_3_, 2.7 mM KCl, 10 mM HEPES pH 7.4) containing either 5 mM Mg^2+^ or 5 mM EDTA, were added to wells and allowed to attach at room temperature for 30 min. The wells were washed with tyrodes (200 μl × 3) and then 150 μl of lysis buffer containing PNP phosphatase substrate (81 mM trisodium citrate, 31 mM citric acid, 0.1 % v/v Triton X-00, 1.85 mg/ml PNP substrate, pH 5.4) was added for 90 min at room temperature. After 100 μl of 2 M NaOH was added and the absorbance read at 405 nm (A_405_) using a Fluostar Optima plate reader (BMG Labtech). Platelet adhesion assays were performed in triplicate and values are reported as means ± standard deviations.

#### HT1080 adhesion and spreading analysis

Collagen films were BSA blocked as for platelet adhesion analysis. 100 μl of HT1080 cells were added at a density of 5 × 10^5^ cells/ml in serum free DMEM containing either 5 mM MgCl_2_ or 5 mM EDTA. After incubation at 37 °C/5 %CO_2_ for 30 min loosely bound cells were removed with 3 × 200 μl PBS washes. Bound cells were detected using the phosphatase substrate as for platelet adhesion.

For spreading analysis, 100 μl of HT1080 suspension at 2 × 10^5^ cells/ml in serum free DMEM were added to BSA blocked films for 60 min at 37 °C/5 % CO_2_. The cells were fixed by the addition of 9 μl of 37 % formaldehyde (final concentration 3.7 %) directly to the cell media. The samples were washed 3 × 200 μl PBS then viewed using a LEICA DMI6000CS phase contrast microscope fitted with a LEICA DFC340FX camera.

#### HT1080 proliferation and film coverage

For measuring HT1080 cell proliferation, collagen films were BSA blocked as for platelet adhesion analysis. 200 μl of HT1080 cells resuspended at 5 × 10^3^ cell/ml in 10 % (v/v) containing DMEM were added to the films. After 1, 2 and 4 days incubation in a humidified incubator at 37 °C/5 %CO_2_ the cells were fixed as for cell spreading analysis. The number of cells per field of view at 20 × magnification were counted using a LEICA DMI6000CS phase contrast microscope in duplicate for triplicates. The results show the mean cells count per field of view ± standard deviations

Film surface coverage was determined by incubating BSA blocked (as for platelet adhesion analysis) collagen films with 200 μl of HT1080 cells at a density of 1 × 10^4^ in 10 % (v/v) serum containing DMEM for 4 days at 37 °C/5 %CO_2_/100 % humidity. After incubation, the cells were fixed and photographed as for cell spreading analysis. The surface occupancy by cells was measured using Image J where the total cell area was divided by the total film surface area to determine the % surface coverage by cells. The data represent mean coverage of duplicate measurements of triplicate samples ± standard deviations.

## Results

### Morphology and dimensional properties of scaffolds

**Fig. 1:**
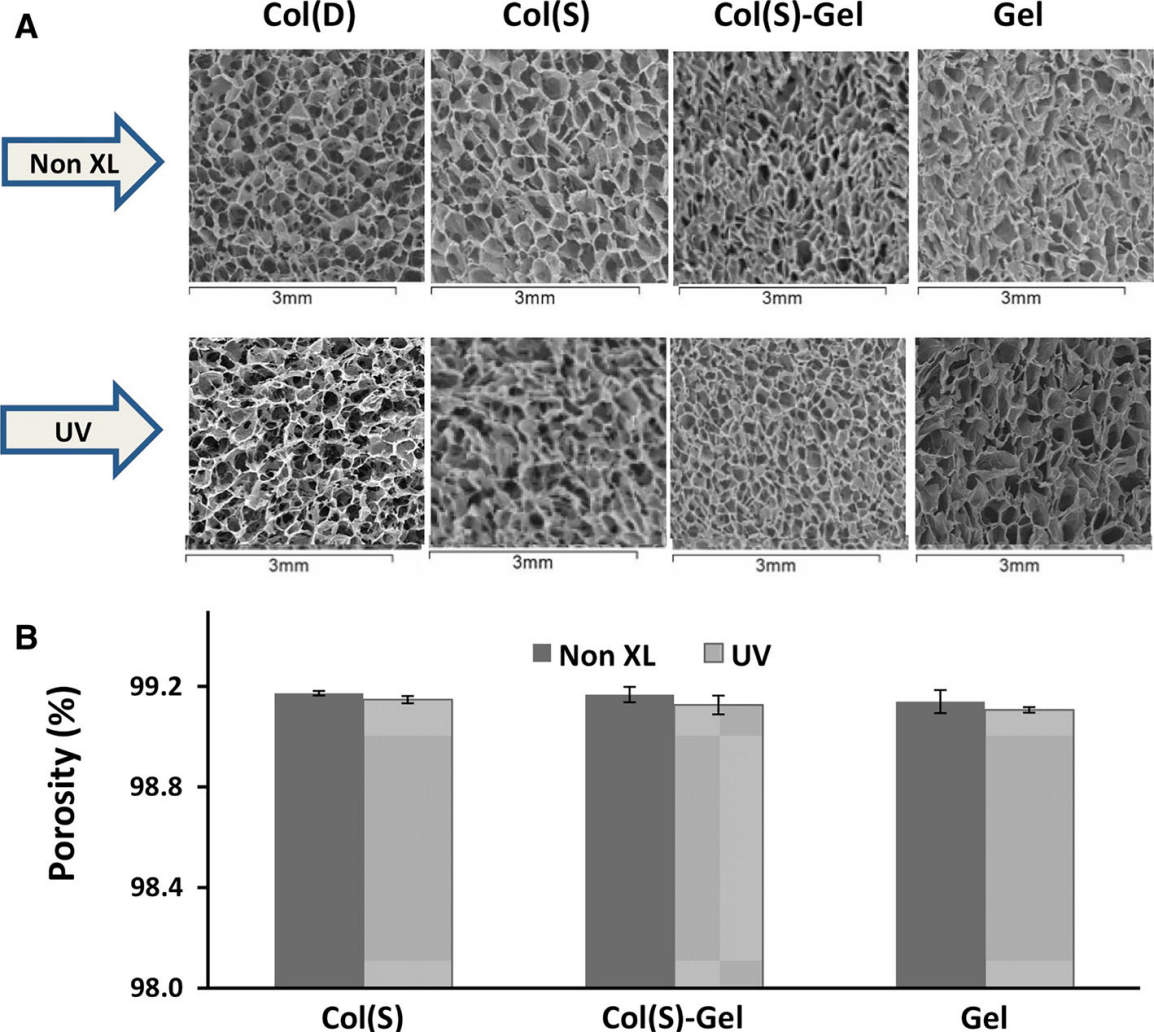
SEM images of non-XL scaffolds of different compositions before and after UV irradiation with an intensity of 0.42 J/cm^2^

SEM analysis of transverse cross-sections of non-XL samples revealed that highly porous scaffolds with homogeneous isotropic inner architecture were obtained for all compositions (Fig. 1, upper images). Images of different longitudinal cross-sections (top, middle and bottom) of non crosslinked scaffolds were also taken and analysed. They showed that the pore architecture was homogeneous in all directions throughout the bulk scaffold. Only limited pore enlargement was present on the top surface and a narrow zone of pore densification on the bottom surface of the scaffold (data not shown, paper in submission)

To assess the possible influence of UV irradiation on scaffold morphology, SEM images of all non-XL and scaffolds before and after UV exposure (intensity = 0.42 J/cm^2^) were taken (Fig. 1).

Figure 1a shows that UV exposure does not introduce any appreciable gross changes to the scaffold morphology for all compositions tested using either Sigma or Devro collagen (S and D respectively). The pore dimensions estimated from SEM microphotographs were predominantly in the range of 130–260 μm, and was independent of the inclusion of gelatin or the collagen source (S or D) selected.

The porosity of scaffolds before and after UV irradiation showed very similar values (99.1–99.2 %) for both S and D Col-based scaffolds and pure Gel matrices (Fig. 1b). These results suggest that UV does not significantly affect scaffold porosity.

### Degradation in water

The objective of this testing was to assess the possible effect of UV irradiation intensity and the nature of Col employed on the dissolution of scaffolds in an aqueous environment. For this, scaffolds were fabricated from Col(S) and Col(D) and irradiated with UV intensities from 0.12 to 0.96 J/cm^2^. These extensive screening studies were carried out in distilled water at pH 7.4 in preference to PBS or DMEM to avoid unnecessary sample handling such as the need to remove salts introduced by such media which could artificially alter the scaffold integrity and so introduce handling error. Our preliminary studies in PBS revealed no significant differences in degradation behaviour of the chemically crosslinked samples. However, for weakly stabilised systems (only UV irradiated or non-crosslinked) the accuracy and reproducibility of results in this biological buffer were lower than in water due to the additional washing procedure.

**Fig. 2:**
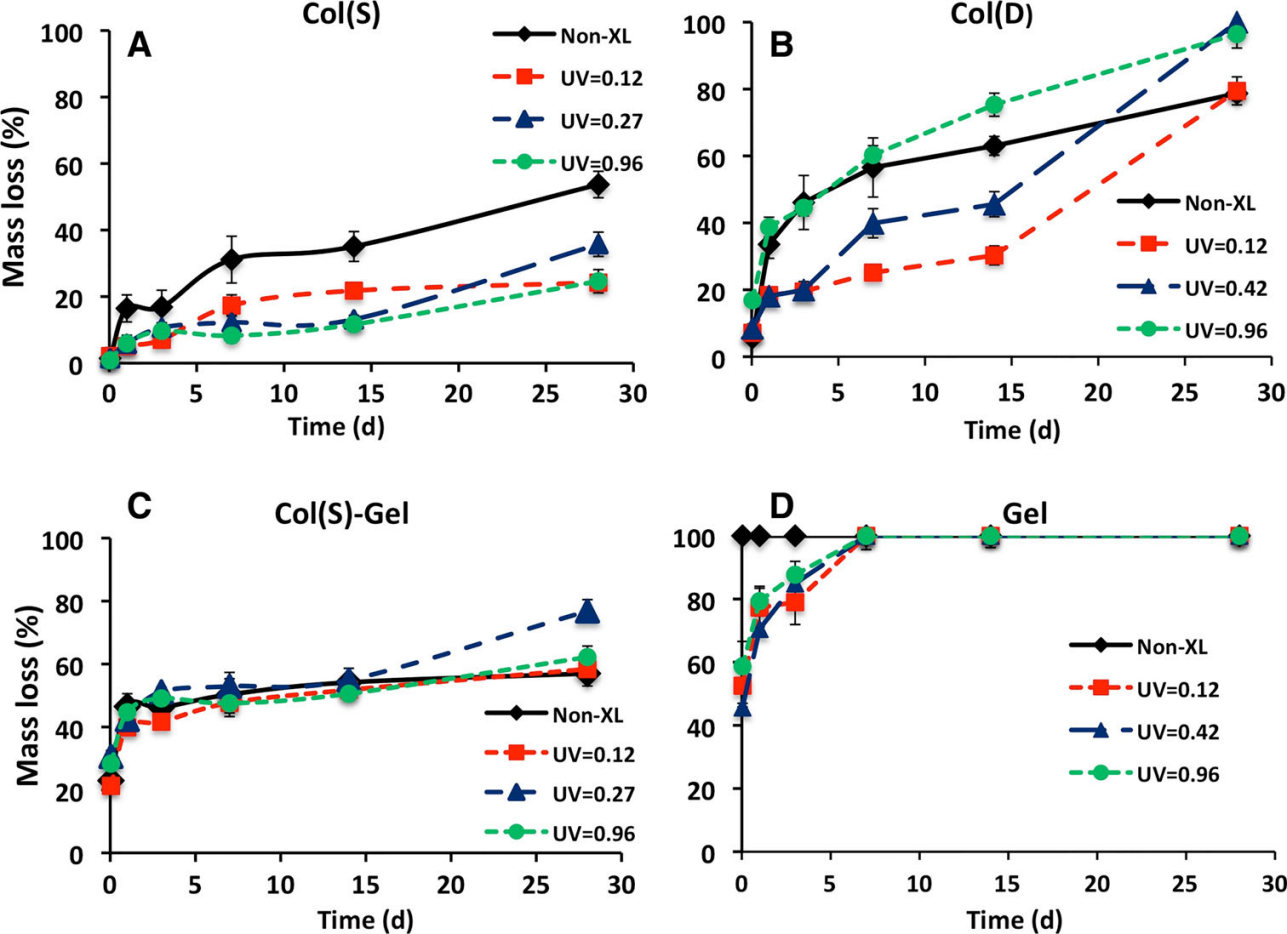
Dependence of mass loss (%) over incubation time (days) on UV intensity for different scaffold compositions, **a** Col(S), **b** Col(D), **c** Col(S)–Gel and **d** Gel. Samples were crosslinked with increasing UV intensity (0—*diamond*, 0.12—*square*, 0.42/0.27—*triangle*, 0.96—*circle* in J/cm^2^

Mass losses (%) of collagen scaffolds obtained from S and D samples, mixed compositions and pure gelatin matrices before and after UV irradiation with different intensities are shown in Fig. 2. The dissolution profiles of pure Col(S) samples indicate that UV light increases their resistance to dissolution up to 28 days in water and that the extent of UV stabilisation was very similar at all intensities employed over all incubation periods (Fig. 2a). For example, the mass loss at an early incubation stage (24 h) decreases from 16 % (Non XL) to 5 and 6 % for 0.12 and 0.96 J/cm^2^ UV irradiated scaffolds respectively. At 28 days UV stabilisation was still evident where the mass loss decreases from 54 % (Non XL) to 24 % (0.12 J/cm^2^) and 25 % (0.96 J/cm^2^). For Col(D) scaffolds, however, only low intensity UV light (0.12 and 0.42 J/cm^2^) stabilised these matrices in aqueous conditions (Fig. 2b), giving diminished mass loss compared with the non-irradiated control, especially for early stages (24 h–15 days) of incubation. By contrast, UV treatment at high intensity (0.96 J/cm^2^) has no effect on the material stability in water up to 7 days, and instead Col(D) UV irradiated samples exhibited less stability than Non XL matrices (compare solid line with dashed for 0.96 J/cm^2^, Fig. 2b) for longer incubation durations. For mixed compositions, very similar results were obtained for Col(S) and Col(D) derived scaffolds. The example of dissolution profiles for mixed Col(S)–Gel scaffolds under UV irradiation is shown in Fig. 2c. From this it can be observed that mass loss was significantly higher for the Col(S)–Gel scaffold than for the pure collagen scaffold presumably due to Gel contribution to overall degradation. For pure Gel samples (Fig. 2d) the UV stabilisation effect was evident for all UV irradiated samples but only at the early incubation period (up to 3 days) after which UV treated Gel samples were completely dissolved.

**Fig. 3:**
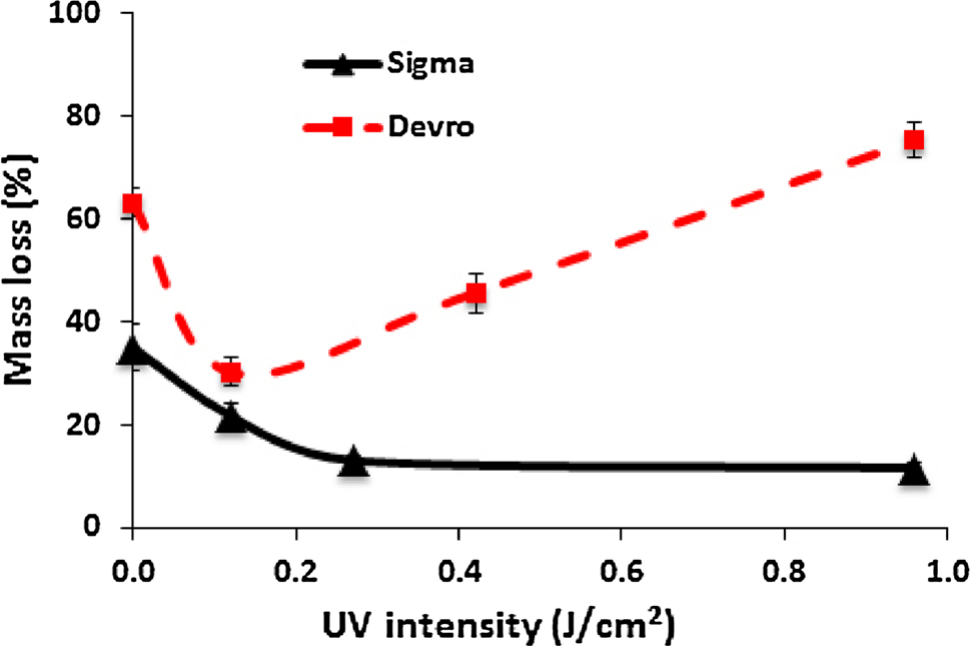
Comparison of the dissolution profiles of Sigma and Devro collagen scaffolds. Mass loss (%) of Sigma and Devro Col UV treated scaffolds after 14 days in water

Comparison of the dissolution properties of collagen scaffolds for the two extreme conditions, (i.e., non-irradiated Col (S and D) and samples exposed to the highest UV intensity (0.96 J/cm^2^), revealed that Non-XL Col(S) matrices are much more resistant to dissolution than Non-XL Col(D) samples (Fig. 2a, b). Additionally, at the highest UV intensity Col(S) and Col(D) scaffolds are affected by UV crosslinking in an opposing manner. This is evident from the observation that UV irradiation significantly enhances Sigma-based scaffold stability in water over all incubation periods but conversely decreased the stability of Col(D)-derived scaffolds at prolonged incubation time. This is highlighted by plotting mass loss against UV intensity after 14 days of incubation (Fig. 3) which shows that lower UV intensities contribute to material stabilisation for both Col(S) and Col(D) based scaffolds, decreasing their dissolution to near convergence at I = 0.12 J/cm^2^. Instead at higher intensities the dissolution profiles of Col(S) and Col(D) based scaffolds follow the opposite tendencies where dissolution decreased with increasing UV intensity for Col(S) but increased for Col(D).

### Amine group content and UV exposure

**Fig. 4:**
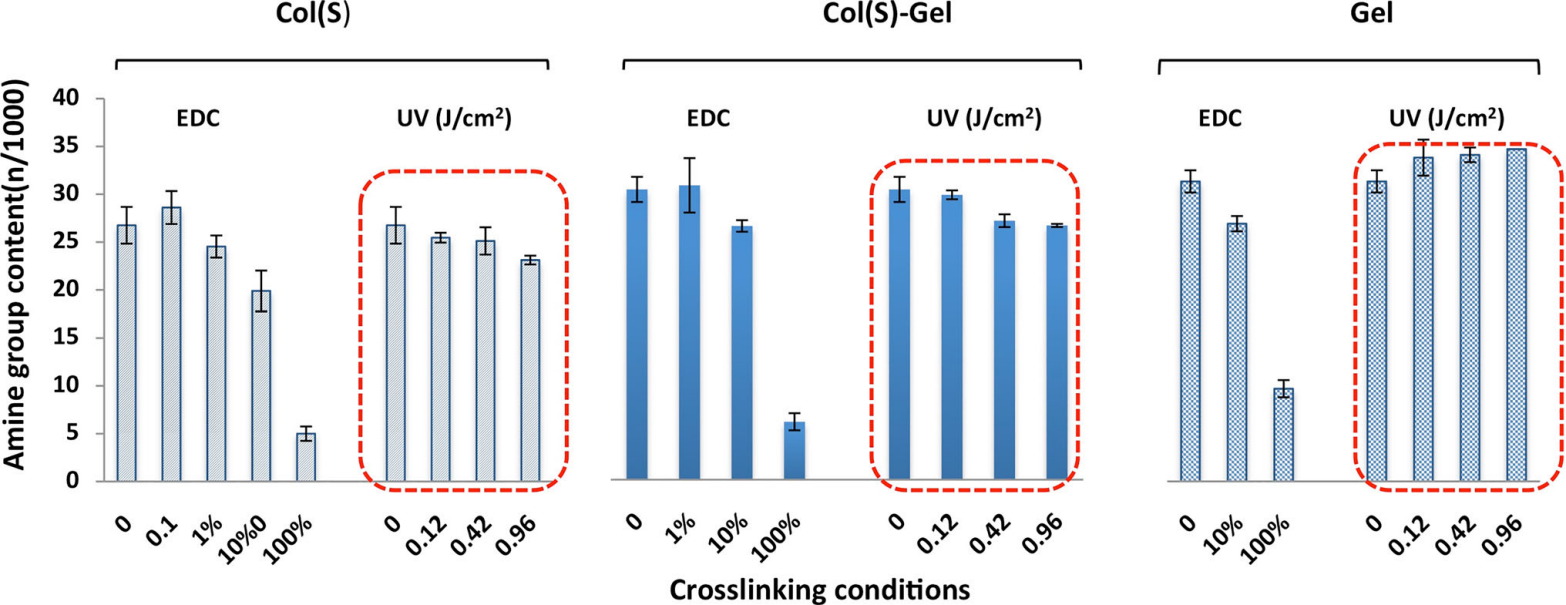
Comparison of the influence of EDC treatment with different concentrations and UV irradiation at different intensities (J/cm^2^) on the free amine group content of Col(S), Col(S)–Gel and Gel scaffolds

In our previous work, we demonstrated that chemical crosslinking, using EDC/NHS treatment at different concentrations, significantly decreases the number of free amine groups remaining on scaffolds composed of both collagen types [54]. For comparison, elements of these results are reproduced in Fig. 4 where 100 % EDC corresponds to EDC concentrations of 11.5 mg/ml, EDC/NHS/COO^−^(Col) = 5/2/1. The present study aimed to establish if UV irradiation similarly affects the amount of free amine groups on Col, mixed Col–Gel and Gel scaffolds (Fig. 4). The results were very similar for scaffolds obtained from both collagen suppliers (S and D) and so for clarity, only Col(S) are shown in Fig. 4 and Col(D) are not shown. These data indicate that very similar amino group content was detected for all scaffold compositions either with or without UV exposure (highlighted areas) suggesting that UV crosslinking does not utilize free amine groups for bonding.

### Mechanical testing of different scaffold compositions

Compressive properties are of great interest when studying the impact of scaffold mechanics on cellular activity. This is because cells, through their mechano-transductive action, tend to bend and buckle individual struts within the scaffold [22, 31, 45]. Therefore the linear elastic (Young’s) modulus (E) calculated here from the stress–strain analysis gives precise measurement of the resistance of the struts to bending and buckling under compression.

**Fig. 5:**
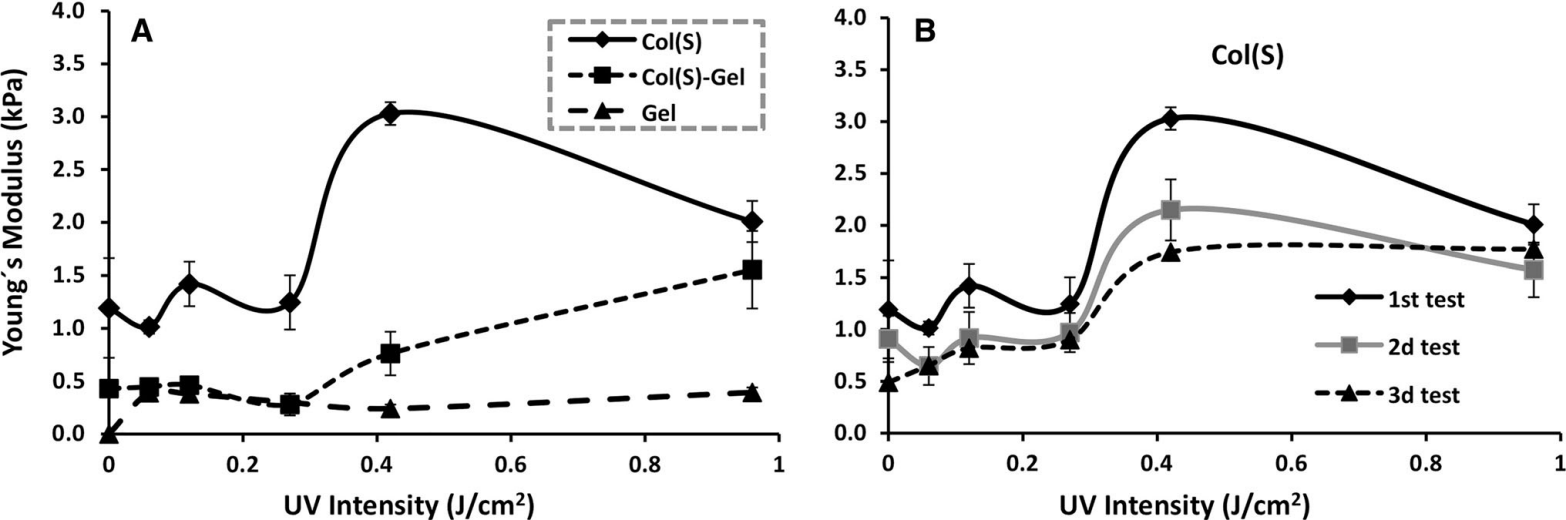
Influence of UV intensity on the compressive modulus (E*) of scaffolds composed of Col(S) (*diamond*), Col(S)–Gel (*square*) and Gel (*triangle*) (**a**). UV effect on successive compressions of Col(S) UV-irradiated scaffolds (**b**)

Mechanical testing was carried out on Col(S), Col(S)–Gel and Gel irradiated with increasing UV intensities. Both collagens (S and D) were tested and Col(S) is shown in Fig. 5. The values of E for Col(S)-containing samples demonstrated that UV treatment significantly increases the elastic modulus for both pure Col(S) and mixed Col(S)–Gel compositions (Fig. 5a). A smaller increase in elastic modulus was observed for Gel samples. Although the UV-induced increase in the elastic modulus for pure Gel was small this treatment gave sufficient stability to allow mechanical testing. By contrast, non cross-linked Gel samples were completely dissolved during the 1 h pre-incubation in water prior to compression testing and so were assigned a modulus of 0.

From this data the elastic modulus of Col(S)-containing scaffolds depend on the intensity of UV light employed. Both pure Col(S) and mixed scaffolds showed no significant differences in the Young’s modulus at intensities lower than 0.27 J/cm^2^ whereas increasing UV beyond this threshold increases the Young’s Modulus of both Col(S) and Col(S)–Gel scaffolds but to differing degrees. For example the elastic modulus of Col(S)–Gel scaffolds increased dose-dependently with UV intensity to a maximum value at the highest UV intensity (0.96 J/cm^2^) tested, whereas for pure Col(S) the maximum elastic modulus was at a UV intensity 0.42 J/cm^2^ (Fig. 5a).

To assess the scaffold behaviour under successive compressions the same sample specimens were compressed three consecutive times. Young’s moduli calculated from the curves after successive compressions for UV treated Col(S) at different intensities (Fig. 5b) showed that although high UV intensity promoted an increase in the Young’s Modulus this was more evident on the first compression. This reduced upon second and subsequent compressions. For example a ~33 % reduction in the elastic modulus was noted on the second versus the first compression for Col(S) irradiated at I = 0.42 J/cm^2^. For Col(S)–Gel scaffolds treated with 0.96 J/cm^2^ the reduction of elastic modulus was ~50 % (data not shown). These results suggest that UV light possesses the capacity to form a limited density network within polypeptide molecules and that UV-induced bonding is not strong enough to resist successive compressions in a wet environment.

Col(D)-containing untreated and UV-exposed samples were also tested under compression. It was found that (a) the elastic modulus of Non-XL Col(D) samples was significantly lower than that of Col(S) scaffolds: 0.55 kPa versus 1.2 kPa, for Col(D) and Col(S), respectively and (b) that UV light does not introduce any appreciable increase in Col(D) scaffold resistance to compression (data not shown). For mixed non treated Col(S or D)–Gel compositions almost identical elastic moduli were obtained independent of the type of Col utilised giving ~0.48 kPa for both Col(S)–Gel and Col(D)–Gel scaffolds. There was also little effect form UV irradiation on the mechanical properties of Col(D)–Gel scaffolds at all studied intensities (data not shown) suggesting that UV light could not provide any additional resistance to compression for Col(D)-derived matrices.

### Synergetic effect of glucose and UV light on scaffold stabilisation

Glucose incorporation was analysed as a method to enhance the efficiency of UV-based crosslinking. Scaffold dissolution in water and the compressive properties of glucose-containing samples, irradiated at 0.42 J/cm^2^ were undertaken. As controls, non-irradiated scaffolds (with the same glucose content) and samples without glucose, but exposed to 0.42 J/cm^2^ UV were included.

#### Dissolution in water of scaffolds with glucose

**Fig. 6:**
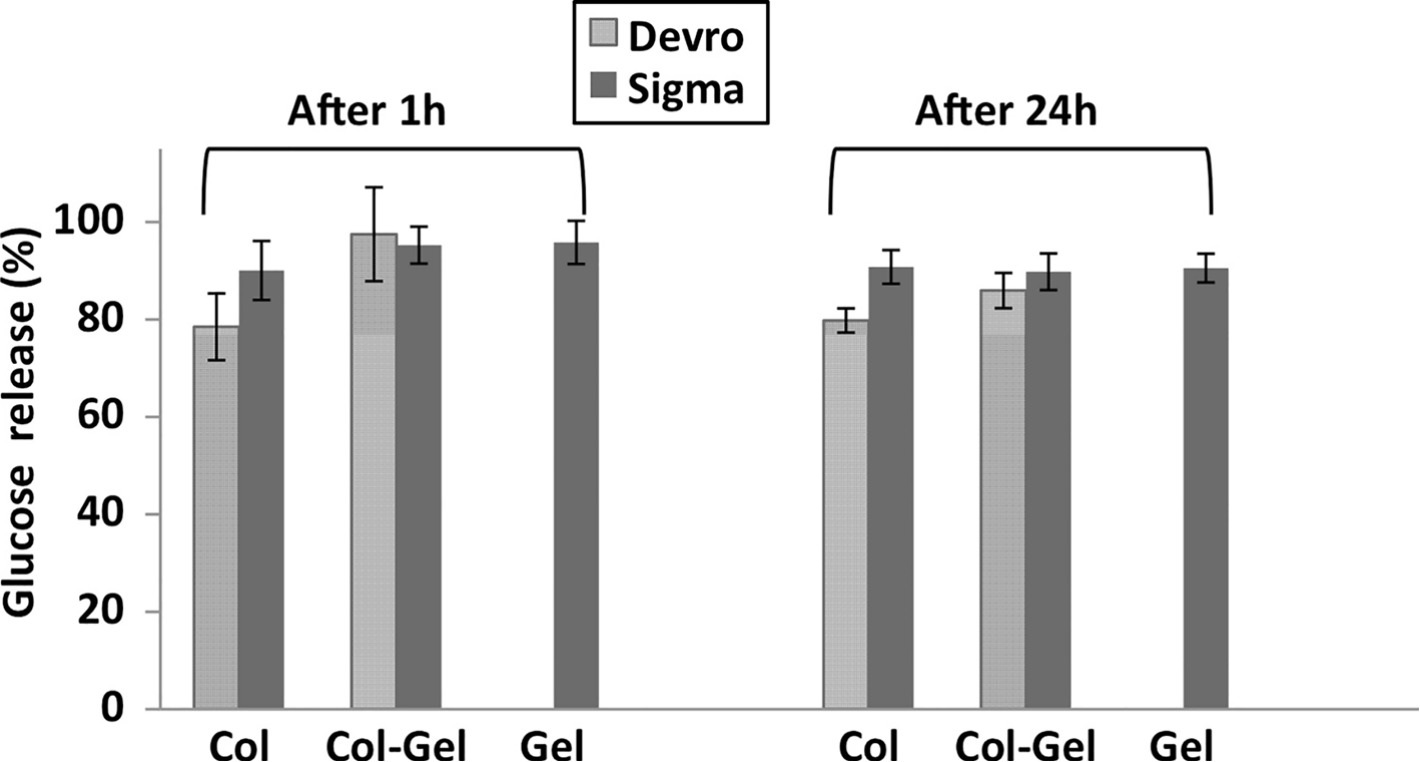
Percentage of glucose release from scaffolds to water after incubation for 1 and 24 h

**Fig. 7:**
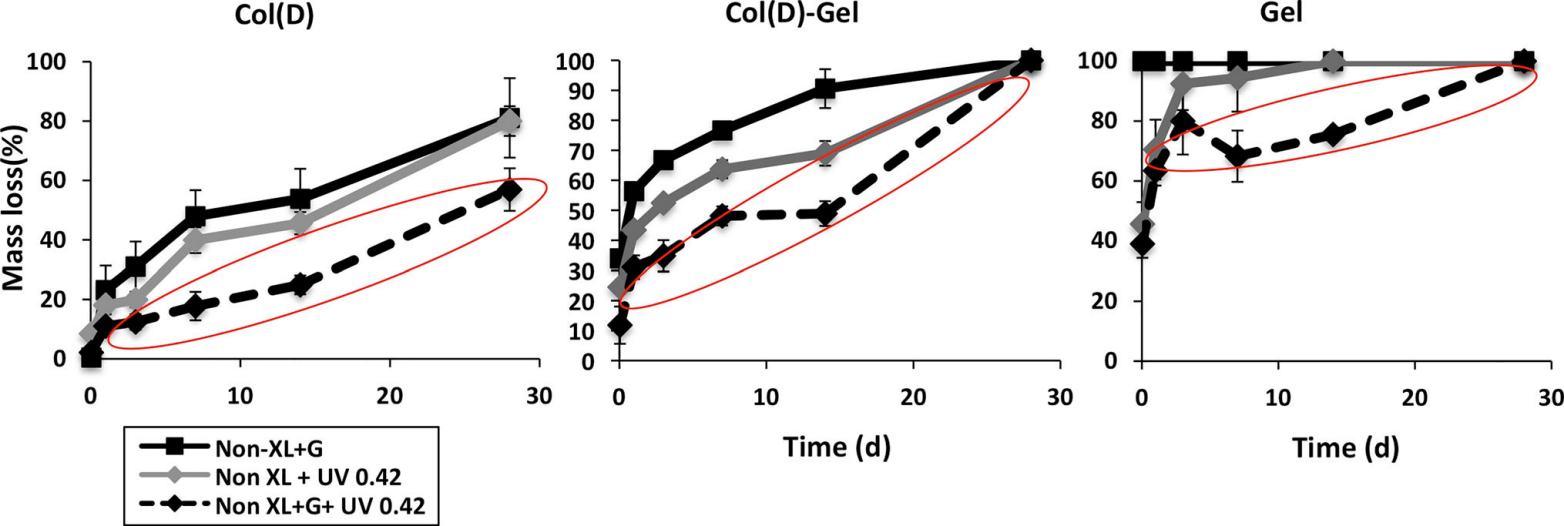
Combined effect of glucose and UV light (0.42 J/cm^2^) on dissolution resistance of Col(D) (*left*), Col(D)–Gel (*middle*) or Gel (*right*) scaffolds

The release of glucose from the glucose-incorporated scaffolds into the bathing solution was measured using a specific, reproducible and sensitive enzymatic assay [53]. This allows for correction of the values of mass dissolution from glucose containing scaffolds to compensate for glucose, rather than Col or Gel mass loss. It was found (Fig. 6) that almost all of the glucose content was released to the media after 1 h of incubation in water independent of scaffold composition and Col type. These values were used then to calculate the contribution of glucose loss to the overall scaffold mass loss to allow estimation of the mass loss of the Col or Gel from the glucose containing scaffolds. Dissolution profiles of scaffolds with glucose either with or without UV irradiation (black continuous and dashed lines, respectively) and UV irradiated scaffolds without glucose (grey lines) are shown in Fig. 7. The collagen with the lower stability, Col(D) was chosen for this study in order to highlight any glucose mediated enhancements in cross-linking.

Significantly lower values of mass loss (highlighted results on Fig. 7) were observed for all glucose-containing scaffolds after UV treatment. These results strongly suggest a synergetic effect of glucose and UV on increased scaffold stability in water.

#### Influence of presence of glucose on compression modulus of UV irradiated scaffolds

**Fig. 8:**
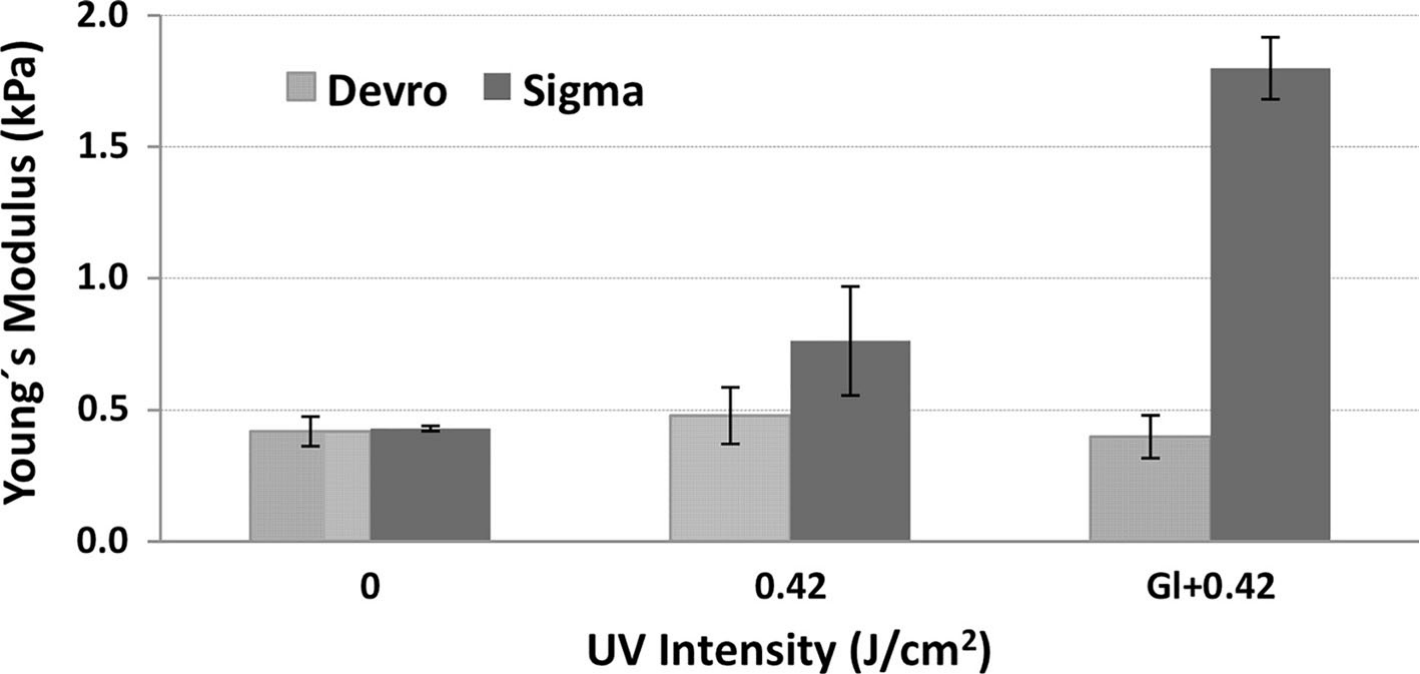
Influence of the presence of glucose on the compressive modulus (E*) of mixed scaffolds composed of Col(S) and Col(D)

Mechanical testing was carried out on Col(S), Col(D), Col–Gel and Gel with and without glucose and with 0.42 J/cm^2^ UV treatment. This irradiation intensity was selected as it provides the maximum value of elastic modulus for Col(S) samples (Fig. 5). The Young’s modulus presented in Fig. 8 revealed that Col(S)-based, UV irradiated matrices, possess significantly elevated compressive properties in the presence of glucose. By contrast the Young’s Modulus of Col(D), UV treated, scaffolds was not affected by the incorporation of glucose. Glucose incorporation achieved very little increase in the mechanical stability of UV crosslinked, Gel scaffolds with the Young’s modulus increasing from 0.24 to 0.33 kP with glucose addition. Therefore a clear synergetic effect between glucose and UV was observed only for Col(S)-derived scaffolds, while for Col(D)-based scaffolds it appears that neither UV light alone nor a combination with glucose could produce further resistance to compression

### Influence of UV irradiation on cell attachment, spreading and proliferation on Col and Gel films

Cells bind to native collagen through collagen specific integrin receptors, for example α_2_β_1_ on the cell membrane. To assay for integrin α_2_β_1_-mediated adhesion the degree of platelet adhesion to Col(S), Col–Gel and Gel films was measured. For this analysis the films were prepared directly into 96-well plates and then treated with 0.42 and 0.96 J/cm^2^ UV light to mimic the 3D struts. These UV intensities (the middle and the highest values of studied interval) were used in the assessment of the amine group content and the other tests reported in this work. Moreover, I = 0.42 J/cm^2^ provides the best mechanical stabilisation for Col(S) matrix.

**Fig. 9:**
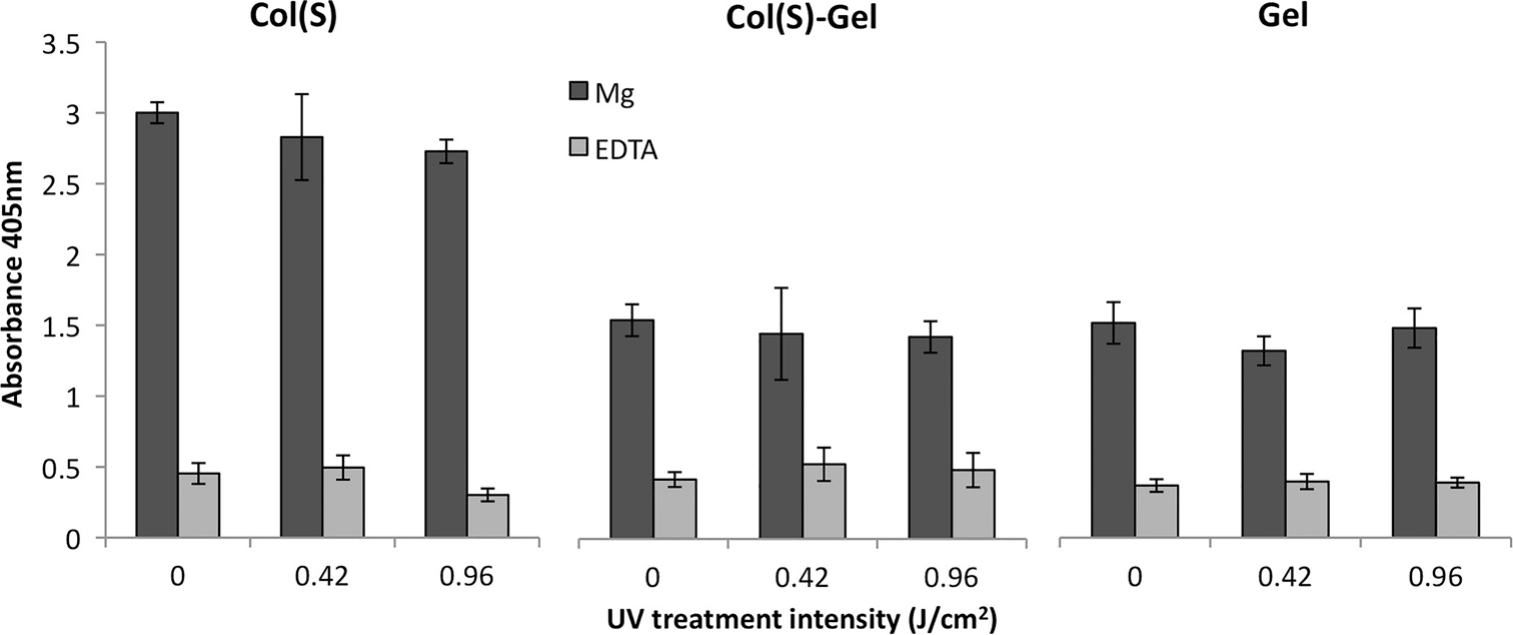
Platelet adhesion to Col(S) (*left*), Col(S)–Gel (*middle*) or Gel (*right*) films irradiated with 0, 0.42 or 0.96 J/cm^2^ UV. Platelet adhesion was measured in the presence of 5 mM MgCl_2_ (*dark grey*) or 5 mM EDTA (*light grey*)

**Fig. 10:**
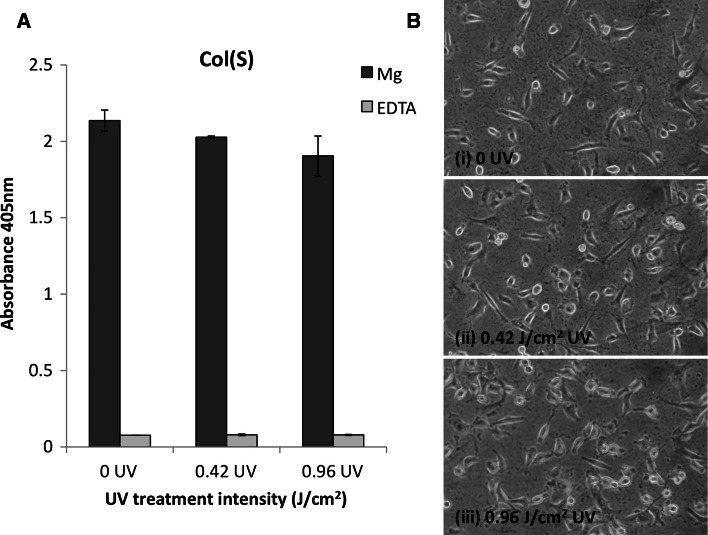
HT1080 attachment (**a**) or spreading (**b**) to Col(S) films irradiated with 0, 0.42 or 0.96 J/cm^2^ UV. Cell attachment was measured in the presence of 5 mM MgCl_2_ (*dark grey bars*) or 5 mM EDTA (*light grey bars*). Cell spreading was measured in DMEM without additional MgCl_2_

Platelets can adhere to collagen through integrin α_2_β_1_ but also a non-integrin receptor (GPIV). As integrin-mediated binding is divalent cation dependent, platelet binding was measured in the presence of magnesium (Mg^2+^) to assess integrin mediated binding and in the presence of the cation chelator EDTA to evaluate GPIV mediated binding (Fig. 9). On Col(S) films, the degree of integrin mediated (Mg^2+^) platelet adhesion was not influenced by 0.42 or 0.96 J/cm^2^ UV crosslinking. Additionally little platelet adhesion was observed in the presence of EDTA showing that integrin-mediated platelet adhesion to Col(S) based scaffolds is not sensitive to UV treatment. Platelet adhesion to Col(S)–Gel and Gel films was lower than observed on Col(S) films. UV had no effect on the level of integrin promoted (Mg dependent) platelet adhesion to either Col(S)–Gel or Gel based scaffolds. Therefore these data indicate that the loss of GXOGEX motifs (by introducing Gel to Col) concurrent with the addition of RGD motifs alters the platelet reactivity of the composite Col(S)–Gel protein films. As gelatin incorporation reduced the level of integrin mediated platelet adhesion the Col(S)–Gel and Gel combinations were not further analysed. Instead Col(S) alone was used to determine the influence of UV crosslinking on HT1080 fibrosarcoma cell adhesion. HT1080 cell adhesion to Col(S) was not influenced by 0.42 or 0.96 J/cm^2^ UV treatment regimens (Fig. 10a). This cell adhesion was dependent upon the presence of Mg^2+^ as little cell adhesion was observed in the presence of EDTA. As integrin α_2_β_1_ is the predominant collagen binding integrin on HT1080 cells, these data indicate that integrin α_2_β_1_ mediated cell adhesion is not influenced by UV treatment of the Col(S) film. Furthermore HT1080 cell spreading was not affected by 0.42 or 0.96 J/cm^2^ UV treatment of Col(S) films (Fig. 10b). For all UV treatments the cells were flattened, phase dark and possessed cellular projections within 1 h of incubation on the Col(S) film surface. It should be noted that cell adhesion and spreading were observed in the absence of serum in the cell media to prevent cell adhesion to serum containing proteins such as vitronectin and fibronectin. Together these data show that integrin α_2_β_1_ mediated cell engagement with Col(S) is not influenced by UV treatment up to 0.96 J/cm^2^.

**Fig. 11:**
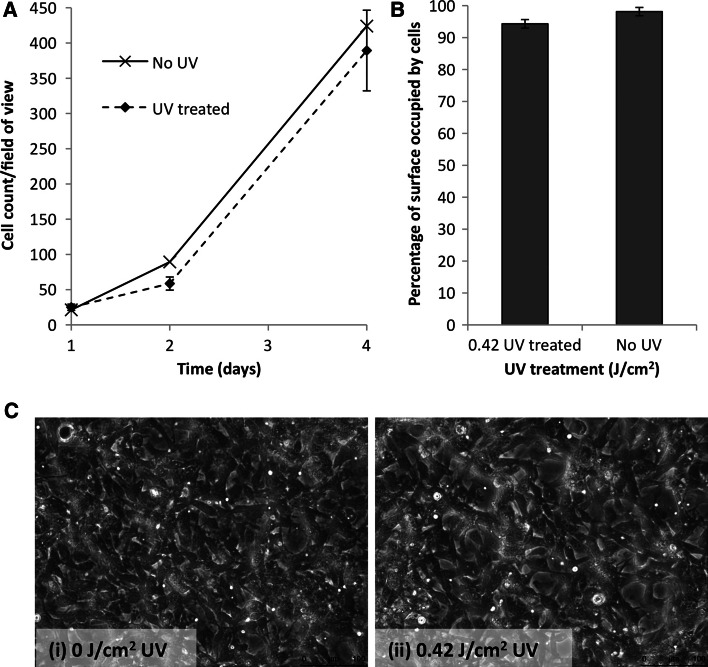
HT1080 cell proliferation (**a**) and cell coverage (**b**, **c**) on Col(S) films irradiated with 0 or 0.42 J/cm^2^ UV. Cells were cultured on the films in DMEM containing 10 % serum. For **b** and **c** cells were cultured for 4 days prior to analysis

To further analyse the cellular response to UV crosslinked Col(S) films, a 0.42 J/cm^2^ UV treatment intensity was employed. This intensity was chosen as it gave optimal dissolution resistance to scaffolds (Fig. 3b). When cultured on Col(S) films HT1080 cells proliferated over a 4 day culture period (Fig. 11a). No difference was noted in the degree of HT1080 cell proliferation on untreated or 0.42 J/cm^2^ UV treated films. Additionally after 4 days in culture the degree of cell colonisation (that is the area occupied by cells) of the films was not affected by UV crosslinking (Fig. 11b). For both untreated and 0.42 J/cm^2^ UV treated films approximately 94–98 % of the film surface was occupied by cells. This is evident from the cell micrographs (Fig. 11c) showing that the majority of both the untreated or 0.42 J/cm^2^ UV treated Col(S) films were populated with a homogenous coverage of cells. Unlike the cell attachment and spreading assays, serum was included in the cell media, being essential to support cell proliferation. Therefore these data show that 0.42 J/cm^2^ UV can be utilised to crosslink Col(S) films without affecting integrin α_2_β_1_ mediated cell attachment, spreading, proliferation or coverage.

## Discussion

Polymer scaffolds for TE must be highly porous, with large surface/volume ratios, to provide sufficient space for cell growth and proliferation [55]. The freeze-drying conditions chosen in this work allowed for the fabrication of 3D scaffolds with an interconnected inner architecture and a porosity of approximately 99 %, a value which falls within the desirable range (>90 %) for efficient cell infiltration. The pore diameter for all scaffolds was in the range 130–260 µm, reported to be suitable for the growth of several cell types including myocytes, endothelial cells and fibroblasts [55–57]. Our SEM studies suggest that Gel incorporation, Col origin (S versus D) and UV irradiation, each have little effect on the scaffold morphology.

The dissolution studies in water showed that UV irradiation could crosslink all of the studied scaffold compositions obtained from both S and D collagens, and that the extent of this process was noticeably dependent on the presence of Gel, the UV intensity and whether Col(S) or Col(D) were employed. In particular, Gel-containing UV treated samples showed significantly higher values of dissolution than pure Col-based scaffolds. This was most likely due to migration of weakly bonded gelatin molecules to the water from their randomly-coiled domain structure. The source of the Col employed also significantly influences the degree of scaffold stability, especially of untreated scaffolds or scaffolds irradiated at higher UV intensities. The amino acid analysis carried out on both samples showed very similar amino acid residue content on both Col(D) and Col(S) (the results are shown in supplementary data). Therefore the differences in the dissolution profiles and mass loss values of Devro and Sigma-derived collagen scaffolds may be attributable to the properties of their macromolecular organisation. Rheological studies and confocal analysis of the architecture of collagen fibres in Col(S) and Col(D) suspensions reported in our previous work [54] revealed that Sigma samples possessed a higher molecular weight with more entangled polypeptide chains than Col(D). This may explain the higher level of dissolution of untreated Col(D) sponges and the different responses of Devro and Sigma collagen scaffolds to UV irradiation. The increase in Col(D) scaffold dissolution at high UV intensities suggests that the more fragmented macromolecular chains of Col(D) are more susceptible to denaturation under UV irradiation, especially at these higher, 0.96 J/cm^2^ intensities. Such denaturation effectively cancels the stability provided by UV induced crosslinking on Devro collagen, and contributes to increased dissolution of its scaffold struts. This supposition is in agreement with reports [34, 35] showing that UV light could lead to denaturation of Col-type molecules with destruction of their triple-helical structure and formation of random-coil domains. It is likely that the longer and more entangled fibrils of Col(S) would be more resistant to UV induced unwinding and to chain scissions. As a consequence, high UV intensity could crosslink Col(S) scaffolds without causing the denaturation effect observed for Col(D). Moreover the differences in dissolution profiles of UV treated Col scaffolds produced from different raw materials may explain the very dissimilar results reported in the literature about the properties of collagenous scaffolds as these were obtained from different suppliers/natural sources.

The observed relatively modest UV cross-linking effect, especially for collagenous materials with less organised macromolecular structures (Col(D) and Gel) arises, in our opinion, from the intrinsic limitation of UV light in forming a high density network within polypeptide molecules. This is due to the relatively small number of aromatic residues (less than 2 %) in collagen molecules [58] which are the major sites for UV-based crosslinking. In turn, this implies that only a small percentage of collagen–gelatin molecules could be linked together through UV-exposure resulting in a loosely crosslinked structure. In addition, collagen fibres in porous matrices may produce an attenuating effect on UV light and so further diminish the degree of cross-linking. UV irradiation could also provoke some conformational changes which together with the denaturation effect might effectively counterbalance the UV stabilising effect of crosslinking. Although UV light alone could not provide a high level of bonding in collagen-type materials, it may be considered as a valuable treatment when moderate enhancement of dissolution resistance is required such as for controlled readsorption of soft tissue applications.

Introduction of glucose to the scaffold composition may be a route to enhancing the efficiency of UV light in stabilising collagen/gelatin-containing scaffolds in aqueous environments. In this work we have demonstrated that the dissolution resistance of all irradiated scaffolds was enhanced after glucose incorporation into scaffold composition. This suggests that glucose-promoted crosslinks formed within Col or Gel molecules under UV light which not only provide additional stability to protein chains but may also prevent unravelling of the collagen triple helix system which, in turn, decreases denaturation.

Compression studies conducted in this work revealed that UV crosslinking conditions, together with scaffold composition and the source of Col employed, have significant impact on the compressive modulus (E*) of the resultant 3D matrices. It is known [22] that differences in the Young’s moduli of 3D sponges with the comparable relative density and similar cell geometry are predominantly attributed to the differences in the elastic modulus of their struts (solid from which sponges are made). As no changes in morphology and dimensional properties were found after UV treatment for all compositions it is logical to assume that the increase in strut stiffness after irradiation is a result of increased UV induced bond formation within the polypeptide chains. Incorporation of glucose to Col(S)-derived samples further increases their resistance to compression after UV irradiation. Instead the lack of perceptible increase in the Young’s modulus of UV treated Col(D) scaffolds (with and without glucose) may be the result of insufficient UV-induced bonding or potentially due to the previously described induced denaturation effect.

It should be mentioned that control of scaffold mechanics is an important issue in the development of 3D cell delivery supports. There are many reports detailing the effect of substrate mechanical properties on cell adhesion and spreading [59–63]. Additionally stem cells have been found to differentiate in response to matrix elasticity, and cells such as cardiomyocytes respond to materials whose stiffness closely resembles that of the developing myocardium. Therefore it is extremely important to ensure that the matrix mechanics closely mimics the in vivo microenvironment in which the cells would normally reside [63, 64]. The results obtained in this work contribute to the knowledge of how the scaffold mechanics could be adjusted and controlled by changing the composition (from Col to Gel) and crosslinking conditions (UV intensities). Incorporation of glucose into the scaffold structure and variation of the Col origin provide further opportunities for tuning the final properties of Col-based scaffolds to a specific in vivo or in vitro application.

Measurement of the amount of free primary amine groups on collagen/gelatin-based scaffolds revealed that UV exposure does not decrease the number of amine groups present. This may be explained by the fact that unlike chemical crosslinking, UV-promoted bonding does not involve reactions between amino acid side chains containing carboxylate (Asp, Glu) and amine (Lys, hydroxylysine) but instead arises from the formation of free radicals on UV-absorbing aromatic groups (Phe, or more rarely, Tyr) in the protein molecules that subsequently attack and form covalent bonds with nearby organic structures. This contrasts to EDC/NHS based treatment which significantly decreases the amine group content as shown in our previous work [54]. As such UV crosslinking should not utilise the acidic or basic groups that are required for physiologically relevant, integrin-mediated cell interactions with collagen, such as through the GFOGER motif. This is supported by our observation that increasing UV crosslinking did not influence platelet and HT1080 cell binding. Furthermore these cell interactions required the presence of the divalent cation magnesium, indicating integrin involvement. As HT0180 cells and platelets predominantly utilise integrin α_2_β_1_ for collagen I based interactions we conclude that UV irradiation does not disrupt sufficient integrin α_2_β_1_ ligation sites to ablate cell binding. It should be noted that we cannot exclude the possibility that a limited proportion of integrin α_2_β_1_ binding sites are denatured by UV treatment. This is in agreement with the limited amide group loss observed on Col(S) after irradiation using the maximal UV dose tested. Instead we assert that the degree of adhesive motif disruption is below the threshold that influences cell behaviour.

When cells receive appropriate cues from their surrounding extracellular environment they will adopt spread morphology and subsequently proliferate. HT1080 cell spreading and proliferation were not affected by UV irradiation of a Col(S) substrate. This indicates that HT1080 cells receive sufficient biological cues from UV irradiated collagen to support full cellular function. The retention of bioactivity is exemplified by full occupancy of a 0.42 J/cm^2^ UV irradiated Col(S) film over a 4 day time course.

The dissolution, mechanical and cell adhesive data presented here support UV treatment as a method to crosslink Col(S) based scaffolds whilst maintaining the physiologically relevant, biological function of the native collagen molecule. Therefore, UV crosslinking presents a clear advantage over chemical crosslinking regimes, such as EDC, in that UV exposure does not diminish important cell recognition sites and consequently retains the scaffold bioactivity for cells.

## Conclusions

This work has defined how UV treatment, the collagen and gelatin composition and the collagen origin (Devro versus Sigma) contribute to the dissolution kinetics and the mechanical behaviour of 3D freeze dried scaffolds. The efficiency of UV irradiation, over a wide range of intensities, as a means of scaffold cross-linking has been evaluated showing that this method is suitable for scaffold stabilisation. Stabilisation in an aqueous environment depends upon both the nature of the collagen-based material employed and the UV intensity, with scaffolds made from the most stable materials showed the greatest stability after UV irradiation. In each case, it would seem that the levels of cross-linking are relatively low. Scaffolds made from pure collagen from different sources have differ in their optimum dose suggesting altered balances between stabilisation from cross-linking and destabilisation from denaturation. The addition of glucose into the scaffold formulation increases the effect of the UV irradiation. As hypothesized, UV cross-linking treatment does not affect cellular attachment, spreading and proliferation on collagen scaffolds. UV irradiation is therefore a method which can be used to provide relatively low level cross-linking of collagen without loss of biological functionality.

## Electronic supplementary material

Supplementary material 1 (DOCX 16 kb)
