# Supplementary material for: Optimisation of UV irradiation as a binding site conserving method for crosslinking collagen-based scaffolds
Source: J Mater Sci Mater Med. 2015 Dec 16;27:14. doi: 10.1007/s10856-015-5627-8 (PMC4681752; doi:10.1007/s10856-015-5627-8)
Supplement: Supplementary file 1 — Supplementary material 1 (DOCX 16 kb) [file 10856_2015_5627_MOESM1_ESM.docx]

Both Col samples (D) and (S) were submitted to amino acid analysis carried out at the Protein & Nucleic Acid Chemistry Facility (Biochemistry Department, University of Cambridge, UK) using a ninhydrin ionexchange analyzer (Biochrom 30) and quantified using Chromeleon software as adapted from (Spackman, D.H., Stein, W.H., Moore, S. *Ana Chem*. 1958;**30**(7):1190-206).

Table Amino acid analysis of Col(D) and Col(S)

| Amino Acid Residue | Col(D) | | Col(S) | |
| --- | --- | --- | --- | --- |
|  | µmole/mg | µg/mg | µmole/mg | µg/mg |
| Asp | 0.41 | 47.3 | 0.41 | 47.3 |
| Thr | 0.15 | 14.7 | 0.16 | 15.8 |
| Ser | 0.29 | 25.1 | 0.27 | 23.7 |
| Glu | 0.58 | 75.4 | 0.56 | 72.4 |
| Gly | 2.85 | 162.6 | 2.71 | 154.6 |
| Ala | 0.95 | 67.6 | 0.94 | 66.6 |
| Val | 0.20 | 20.1 | 0.21 | 20.5 |
| Met | 0.04 | 5.3 | 0.05 | 6.2 |
| Ile | 0.11 | 11.9 | 0.11 | 12.7 |
| Leu | 0.22 | 24.6 | 0.23 | 26.0 |
| Tyr | 0.04 | 6.0 | 0.04 | 7.0 |
| Phe | 0.11 | 16.9 | 0.11 | 16.7 |
| His | 0.04 | 6.1 | 0.04 | 5.7 |
| Lys | 0.23 | 29.3 | 0.19 | 24.4 |
| Arg | 0.42 | 65.4 | 0.42 | 65.7 |
| Pro | 1.02 | 99.5 | 0.97 | 94.6 |
| HO-Pro | 0.74 | 83.6 | 0.69 | 77.6 |
| Total: |  | 761.4 |  | 737.3 |
